# Multiscale modeling of lithium ion batteries: thermal aspects

**DOI:** 10.3762/bjnano.6.102

**Published:** 2015-04-20

**Authors:** Arnulf Latz, Jochen Zausch

**Affiliations:** 1 German Aerospace Center (DLR), Stuttgart, Germany; 2 Helmholtz Institute for Electrochemical Energy Storage, Ulm, Germany; 3 University of Ulm, School of Chemistry, Ulm, Germany; 4 Fraunhofer Institute for Industrial Mathematics (ITWM), Kaiserslautern, Germany

**Keywords:** lithium ion batteries, multiscale modeling, heat transport

## Abstract

The thermal behavior of lithium ion batteries has a huge impact on their lifetime and the initiation of degradation processes. The development of hot spots or large local overpotentials leading, e.g., to lithium metal deposition depends on material properties as well as on the nano- und microstructure of the electrodes. In recent years a theoretical structure emerges, which opens the possibility to establish a systematic modeling strategy from atomistic to continuum scale to capture and couple the relevant phenomena on each scale. We outline the building blocks for such a systematic approach and discuss in detail a rigorous approach for the continuum scale based on rational thermodynamics and homogenization theories. Our focus is on the development of a systematic thermodynamically consistent theory for thermal phenomena in batteries at the microstructure scale and at the cell scale. We discuss the importance of carefully defining the continuum fields for being able to compare seemingly different phenomenological theories and for obtaining rules to determine unknown parameters of the theory by experiments or lower-scale theories. The resulting continuum models for the microscopic and the cell scale are numerically solved in full 3D resolution. The complex very localized distributions of heat sources in a microstructure of a battery and the problems of mapping these localized sources on an averaged porous electrode model are discussed by comparing the detailed 3D microstructure-resolved simulations of the heat distribution with the result of the upscaled porous electrode model. It is shown, that not all heat sources that exist on the microstructure scale are represented in the averaged theory due to subtle cancellation effects of interface and bulk heat sources. Nevertheless, we find that in special cases the averaged thermal behavior can be captured very well by porous electrode theory.

## Introduction

The main challenge for establishing an ab initio multiscale simulation approach for batteries or electrochemical storage devices in general is the extremely complex chemical context in which those devices are operated [1]. There is no commercial battery which is produced from pure active materials for the electrodes and from a pure mixture of salt and solvent for the electrolyte alone. Usually, it is necessary to add soot to the slurry for electrode production in order to counteract the poor electronic conductivity of the active materials of negative electrodes. The active material is not used as one block (except in thin film batteries) but is ground into a powder or artificially designed into complex nanostructures in order to increase the available surface for insertion or conversion reactions. To ensure the mechanical stability of the electrode, binder has to be added, which in turn is not without consequences for the electrochemical properties of the batteries. Therefore the nano- and micrometer-scale structure of the battery is as important as the material itself to obtain a “good” electrode, where “good” is defined with respect to the envisioned application and not with respect to the materials properties. Analogous modifications are necessary to obtain a good conducting electrolyte that is stable under high voltages and chemical compatible with the chosen electrode materials. Thus, additives are used in order to enhance the ionic conductivity and to improve the chemical compatibility. Also the properties of the solid electrolyte interphase (SEI) on the negative electrodes, which is essential for the long time stability of a battery [2], are strongly influenced by the composition of the electrolyte. Therefore it would not be possible to design with simulations an optimal electrolyte without considering, e.g., the impact of the electrolyte on the electrochemical reaction kinetics at the interface of electrolyte and active particles or the complex chemical reactions leading to the growth of the SEI, plating and electrochemical reactions initiated in the bulk of the electrolyte at high potentials.

A successful strategy for the development of predictive theories and simulation tools has therefore to guarantee that the theoretical concepts on the different spatial scales from atomistic to cell scale have sufficient overlap to ensure the possibility of a systematic upscaling procedure. This argument addresses the problem of identifying a representative volume element, which is small enough for being able to use the simulation techniques on the fine scale but big enough for the results to be usable on the large scale. This necessary condition requires the development of systematic rigorous theories on each scale from quantum chemistry for the atomistic scale, over particle-based models utilizing classical force fields to continuum theories. But even within continuum theories it is for practical reasons important to distinguish between microstructure-resolved and porous-electrode theories to develop consistent theories for both scales.

The knowledge of the material parameters and their dependency on composition or atomistic structure is the starting point for a rational design of energy storage materials [3]. Density functional theory with all its approximations [4–5] if combined with statistical mechanics methods is in this context the most successful method to simulate material properties of electrochemically active materials [3,6]. The combination with statistical methods is important to bridge the gap between zero-temperature DFT simulations in vacuum and the properties of the studied materials at finite temperatures in contact with different phases. Standard DFT simulations usually concentrate on individual electronic processes without considering the interplay with the environment or competing electronic processes, which might be statistically and thus macroscopically much more significant [7]. In ab initio thermodynamics, DFT is combined with ideas from statistical mechanics in order to obtain the Gibbs free energy of bulk and interfaces at finite temperatures. Especially for the calculation of differences of Gibbs free energies, which are relevant for the determination of stable equilibrium configurations, the accuracy is higher than might be expected from the absolute accuracy of the DFT simulation, which usually contain many simplification and assumptions on the structure of the solution [4–5]. By using cluster expansions [8] it is possible to combine DFT with kinetic Monte Carlo (KMC) simulations to obtain collective diffusion coefficients for lithium ions at concentrations beyond the dilute limit [9]. To obtain the collective diffusion coefficient is crucial since there is a difference between the self-diffusion coefficients and the collective diffusion coefficient [10]. As will be shown below, it is the collective diffusion coefficient that is relevant for the transport of lithium ions in the solid active particle as well as in the liquid electrolyte. The collective diffusion coefficient can be written as a product of a thermodynamic factor, which can be obtained from the chemical potential, and a kinetic coefficient, which is a measure of the energy barriers caused by the local environment of the Li ions. They may depend in solids on vacancy distributions as well as on the local lithium-ion concentration itself [9,11–13]. The diffusion coefficients for liquid electrolytes might more easily obtained from molecular dynamics (MD) simulations once the force fields for the interaction between the molecules are known [14–15].

Information on interface properties can be obtained from MD simulations and from DFT simulations [15]. MD simulations are especially relevant to study the solvation properties of the ions [15–17], which are important to understand contribution of solvation forces to the intercalation kinetics. The actual barriers for intercalation can be addressed by DFT simulations [18].

The change in mechanical properties upon intercalation is very important to understand degradation phenomena in batteries. Usually the change in the behavior of cells can be simulated by using macroscopic continuum models [19–21]. But knowledge of the change in the specific volume of the material as function of Li concentration and the change in the elastic constants is necessary to parameterize the continuum models. This information can be extracted from DFT simulations [22–23]. Phase changes, initiated by the intercalation of lithium ions, require additional information on the stability of phases [24] and interface properties. The evolution of electrochemical active interfaces can be described by phasefield theories [25–26], which provide an approximate continuous description of the dynamics of interfaces [27]. They need as input the interface free energies between the phases. This interface free energy is accessible to DFT simulations [28–29]. On the basis of this information the intercalation properties of phase separating materials can be modeled [30]. Also, predictions for the stress [31] induced by the phase front and its influence on the intercalation dynamics [32] are possible. It should be mentioned that there are still many unsolved issues and not completely consistent predictions for the intercalation properties of phase-changing many-particle electrodes [33–37].

The production of heat in batteries is a very important information for the proper prediction of degradation phenomenon. Since heat is a thermodynamic concept it has to be dealt with on the macroscopic scale. From atomistic simulations the determination of Gibbs free energies, entropies and insertion properties (e.g., kinetic barriers, chemical potential of adsorbed species, solvation energies) are obtained. The relation of these quantities to the actual heat production has to be derived from systematic thermodynamic theories [38–41]. Most of the literature on heat transport in lithium ion batteries uses phenomenological porous electrode theories [42–49], which are not based on a systematically derived thermodynamic consistent theory. In [45], the porous electrode theory is derived with the help of volume averaging applied to the phenomenological pore scale model. Full 3D simulations of thermal effects in electrode microstructures do, to the best of our knowledge, not exist; except for [50] in which the heat sources in a microstructure of a LiCoO_2_ cathode are obtained with the help of phenomenological expressions for the heat sources and the current distribution in the electrode [51].

A general overview about multiscale modeling and simulation strategy, including an overview about available software concepts in the context of electrochemical storage and conversion devices is given in [52].

We will concentrate in our article on the systematic derivation of fully coupled transport equations for ion, charge and heat transport in lithium ion batteries on the nano- and micrometer-scale as well as on the cell scale . The cell-level equations will be obtained from an analytical upscaling procedure to ensure the consistency with pore-resolved theory. Where possible, we will point out the necessary input from ab initio atomistic scale theories. The microscopic as well as the cell-level theory are simulated for a virtual microstructure and its homogenized effective porous electrode description . The comparison of the averaged results of the microscopic simulation exhibit remarkable agreement with the simulation of the porous electrode theory, but we find very strong fluctuations around the average. Especially for the prediction of degradation phenomena these fluctuations might be crucial.

## Non-equilibrium thermodynamics

To make contact with theories on atomistic scales, it is necessary to formulate the continuum theories in terms of quantities that have a well defined physical meaning and can either be obtained by simulations on atomistic scales or from independent experiments. Even if information about energy barriers and reaction rates can be obtained from density functional theory for the system under investigation, additional modeling steps are necessary to obtain the relevant parameters for the kinetic models used in mesoscopic reaction–transport theories [53–54]. Especially the formalism of ab initio atomistic thermodynamics, which combines DFT simulations with strategies from statistical mechanics [3,6–7], allows one in principle to determine Gibbs free energies, reaction rates and relevant transport coefficients for materials used in electrochemical applications. The transport equations on the continuum scale have to be based on the same quantities. Only then, the information obtained from the quantum scale can be transferred to the continuum scale. Very often continuum scale equations are not derived, but formulated on phenomenological grounds. This approach leads to “effect driven” theories, which try to include the known phenomena (e.g., diffusion, electroosmosis, Peltier effect, double layer properties [53]) without considering the possible existence of an underlying coherent theoretical structure. Such a structure may require relations between transport coefficients in order to ensure positive entropy production and may reveal information about the nature of the considered continuum fields, which are essential to make contact to atomistic scale simulations and to experiment or influence the form of the charge distribution in the double layer [27]. Only a rigorous derivation within a systematic theoretical framework can reveal such a structure. In order to demonstrate the impact of the chosen continuum fields on the structure of a continuum theory we re-derive the equations for coupled transport of ions, charge and heat in a lithium ion battery by using the framework of rational thermodynamics [39–40]. This derivation recovers the equation in [41,55] and shows in the isothermal case the equivalence with the seemingly different theory of [27]. The theory is valid for transport on pore-scale-resolved battery structures. The cell-level equations, which are consistent with the derived microstructure theory are then derived by using systematic volume averaging. It is shown that some of the reversible heat sources of the bulk and the interfaces cancel each other in the averaged macroporous theory. The cancelation is also demonstrated by explicitly simulating the coupled transport on the microscale and analyzing all heat sources in the bulk and the interfaces. Since heat sources lead to thermal stress, there are possible sources for degradation on the microscale that cannot be detected on the macroporous scale. The cancelation also demonstrates the importance of a consistent formulation of interface condition and transport equations. The usual “effect driven” procedure, in which bulk equations and interface conditions are formulated completely independent of each other, may easily miss such cancelation effects. A generic starting point for the derivation of the transport equations is a mixture of a positively charged and a negatively charged species, and a neutral component. This mixture is able to represent an electrolyte consisting of a salt, dissolved in a solvent as well as transport of Li ions and electrons in an active particles consisting of a neutral host lattice. Different derivations are necessary for ionic liquids (mixture of positive and negative charges only) and solid electrolytes (ionic conductors). In a liquid electrolyte these are positive cations, negative anions and a neutral solvent. In conventional Li ion batteries under normal operating conditions, mass convection can be excluded as transport mechanism, but will always be a possibility in a systematic theory. Especially, if there are side reactions leading to film growth or convective gas transport after electrolyte degradation, convective transport might be initiated as a consequence. In general, it is sufficient to consider transport driven by electric fields and gradients in concentration, temperature and stress. In our derivation, we follow closely the notations used in [40] and neglect stress gradients for simplicity.

### Transport theory

First, the general transport theory for a bulk system consisting of three interacting species (negative, positive and neutral species) in a electric field will be derived, before we discuss the boundary conditions at the interfaces between different chemical environments (electrolyte and active particles). Starting point of the derivation are the conservation equations for the three mass densities ρ_α_, the momentum **g** and the energy density ε of the system in an electric field. Although we will set the barycentric velocity **v** to zero at the end of the derivation, it is necessary to include at least a stationary velocity field, in order to obtain the coupling to electro-mechanical stresses. The mass conservation of the species can be written in the form

[1]
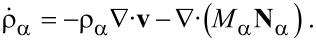


**N**_α_ is the molecular flux and *M*_α_ the molar density (kg/mol) of the species α. The convective or total time derivative 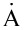 for some variable A is in the usual form given by

[2]
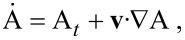


where A*_t_* is the partial derivative ∂A/∂*t* Since the total density 
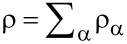
 is conserved, i.e., 
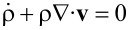
, the requirement

[3]
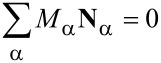


has to be fulfilled. The equation for the momentum density has the very general form

[4]
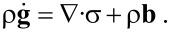


Here **b** is an external force density, σ is the stress tensor and 
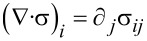
 (the Einstein summation convention for the repeating index is used). The equation for the energy density ε is given by

[5]
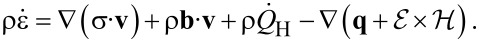



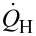
 is the local heat production, **q** is the heat flux, and 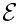 and 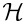 are the Galilei invariant electrical and magnetic fields,

[6]
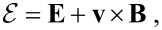


[7]
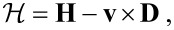


which couple the electric field and magnetic field with the dielectric displacement **D** and the magnetic induction **B**. We may eliminate the force **b** from Equation 5 by using Equation 4 and obtain

[8]
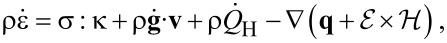


where κ*_ij_* = ∂*v_i_*/∂*x_j_* is the (non-symmetrized) strain rate tensor and 
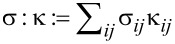
. The Maxwell equations in the Galilei invariant approximation can be written as

[9]
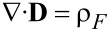


[10]
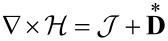


[11]
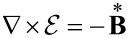


[12]
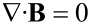


[13]
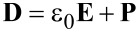


[14]
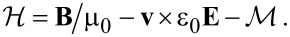


We introduced the Galilei invariant current 
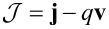
, the magnetization 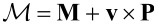 and the flux derivative 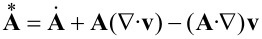. The free charge density ρ*_F_* is related to the molar density *n*_α_ by 
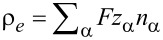
, with *z*_α_ being the charge number (i.e., the multiples of positive or negative elementary charges) and *F* the Faraday number. **P** is the polarization due to bound charges.

In order to derive constitutive equations, we make use of the inequality for total change of the entropy density

[15]
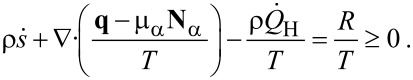


Here *R* is the yet unknown entropy production and μ_α_ is the chemical potential of species α. An expression for μ_α_ will be derived shortly. By eliminating the heat production 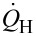 from Equation 15 and some reformulations of 
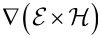
 we obtain

[16]
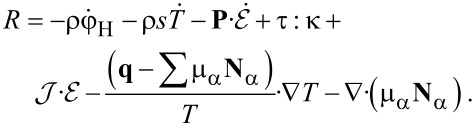


Here φ_H_ is the specific free energy density (with respect to the total mass) and the electromechanical stress tensor is given by (neglecting all contributions from magnetic fields)

[17]
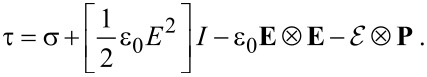


The electromagnetic specific (Helmholtz) free energy, φ_H_, is given by

[18]
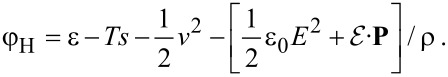


The constitutive equations follow from Equation 16 and the form of the material law for the free energy φ_H_. The influence of magnetic fields on batteries is usually neglected. For the purpose of this article, we are also not interested in the calculation of mechanical strain of active particles. Therefore, the free energy density can be written as

[19]
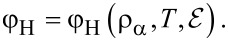


If phase-changing materials are described, this free energy may also be interpreted as free energy functional. For instance, in the case that the free energy also depends on the spatial derivatives of the densities as in phase-field theories for batteries [30,56]. The total derivative of the free energy (Equation 19) is given by

[20]
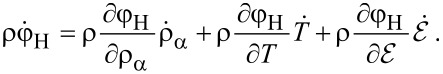


With this and the equation for the free energy, Equation 16 can be transformed into

[21]
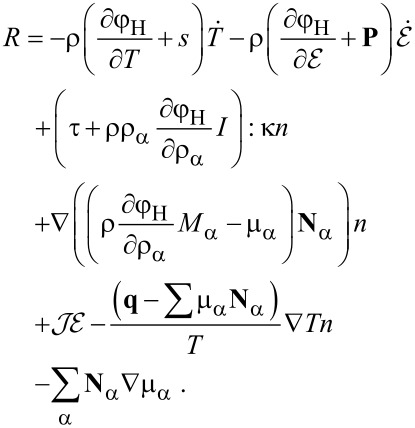


The requirement, that the entropy production *R* of Equation 21 has to be strictly positive leads to the relations

[22]
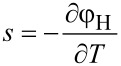


[23]
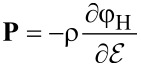


[24]
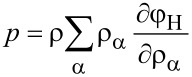


[25]
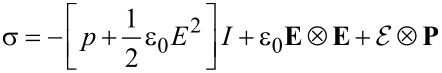


[26]
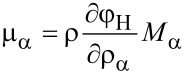


[27]
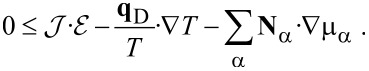


Here *p* is the pressure and σ is obtained from Equation 17 and the necessary condition τ + *pI* = 0 imposed by the positivity of the entropy production *R*. We also introduced the purely diffusive heat flux 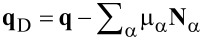. If the velocity is allowed to change in time it can also be shown that the momentum density **g** is given by

[28]
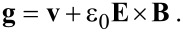


The momentum equation (Equation 4) can be written (by using Equation 4, Equation 25 and Equations 10–14) as

[29]
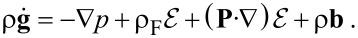


In most of the battery literature the momentum equation (Equation 29) is not used, although it implies, that large gradients in the pressure are to be expected for strong electric fields. This, for example, is the case in the double layer. Since the chemical potentials of the ionic species and the solvent are, in general, dependent on the pressure, they will in turn exhibit large gradients leading to a non-negligible contribution to the variation of the ion concentration [27]. For incompressible systems, i.e., ρ = *const*, the chemical potential depends linearly on the pressure and can be written as function of the pressure and mass or particle fractions *y*_α_ = *n*_α_/*n* [27] with 
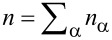
 being the total molar density:

[30]
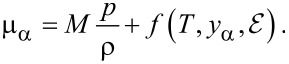


The energy equation (Equation 8) leads to

[31]
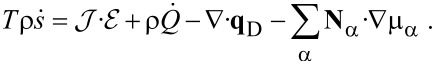


Due to the constraint in Equation 3 there are only two independent fluxes and since the total density ρ can be safely considered to be constant, there are only two independent densities. The form of the constitutive equations depends on the choice of independent variables. This freedom seems to lead to different theories [27,55,57]. Our systematic derivation offers the possibility to formulate the transformation rules between the different set of independent fields, in order to analyze the similarities and differences of the theories.

If we choose as independent densities the molar densities *c*_+_ and *c*_−_, the corresponding fluxes are **N**_+_ and **N**_−_. The electric current **j** is written as **j** = *z*_+_**N**_+_ + *z*_−_**N**_−_ and the electric field as 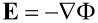. In addition, if we assume that there are no bulk heat sources 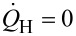 (i.e., no side reactions in the bulk), the resulting transport equations for **v** = 0 and the entropy production are

[32]
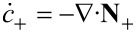


[33]
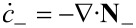


[34]
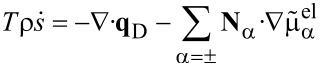


[35]
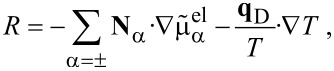


where 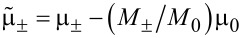 is the effective chemical potential for the ions and anions and 
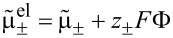
 is the electrochemical potential. To guarantee the positivity of the entropy production, *R*, the fluxes and heat current are written in a form that gives *R* a strictly positive quadratic form. This can be achieved by choosing

[36]
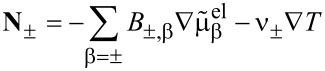


[37]
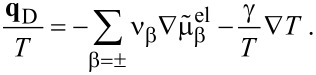


This is the form of constitutive equations used in [27,58] for isothermal systems (i.e., 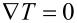). Specifically we obtain the well-known result that the fluxes are proportional to a combination of the electrochemical potentials and in addition to a term proportional to the gradient of the temperature. Choosing the mobility matrix to be a symmetric positive matrix guarantees the positivity of the entropy production for every solution of the transport equations. Specifically we get *B*_++_ > 0, *B*_−−_ > 0 and 
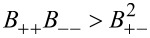
. In dilute solution theory, the mobility *B*_+−_ is set to zero, i.e., 
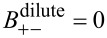
. If temperature variations are included we obtain further conditions on the heat conductivity, λ (λ >0), and the off-diagonal terms ν_±_. Very often the constitutive equations are formulated in terms of chemical potentials 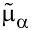, electrical or Galvani potentials Φ. This form can be easily obtained from Equation 36 and Equation 37:

[38]
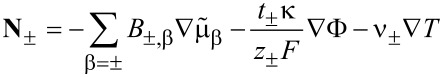


[39]
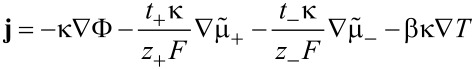


[40]
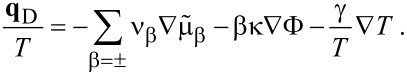


The conductivity κ and the transference numbers are given by the components of the mobility matrix

[41]
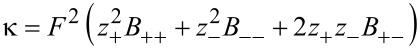


[42]
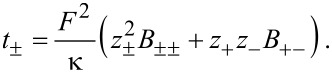


Note that *t*_+_ + *t*_−_ = 1. The Seebeck coefficient β is defined by

[43]
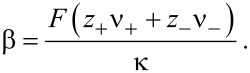


It is related to the Peltier coefficient Π through Π = *T*·β.

Since the ionic fluxes **N**_±_ and the (free) current **j** are not independent of each other, Equation 38 can also be brought into a more condensed form, obtained in [41,55] by using the definitions in Equation 41 and Equation 42 for *t*_±_ and κ and introducing the chemo-electrical potential of the positive ions 
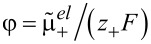
.

[44]
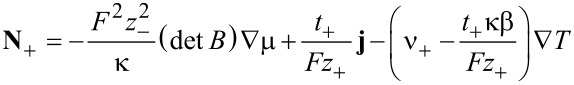


[45]
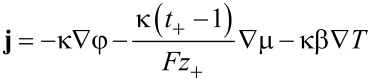


[46]
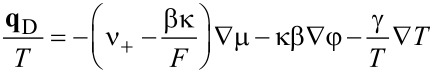


[47]
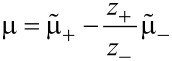


[48]
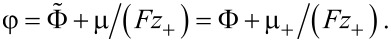


To use φ is the choice in most of the electrochemical literature, naming it usually “electrical potential”. This might be considered as an unfortunate semantic inaccuracy but it has in fact consequences for the relation to free electric charges. The latter are determined by Φ through Equation 10 and not by φ.

It is possible to obtain the formulation in Equation 44 and Equation 45 directly from the entropy law by choosing the flux **N**_+_ and the electric current **j** together with the molar density *c*_+_ = ρ_+_/*M*_+_ =: *c* and the free charge density ρ_F_ = *F*(*z*_+_*c*_+_ + *z*_−_*c*_−_) as primary variables.

In this formulation it can be easily seen as well that the diffusion coefficient *D* at vanishing current **j** is proportional to the determinant 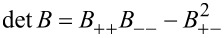 of the mobility matrix *B*. The requirement of positive entropy production mentioned above is therefore the equivalent of having a positive collective diffusion coefficient.

The transport of the anions is ruled by the same diffusion coefficient as the diffusion of cations, which can be easily seen from Equation 44 and Equation 45 and **N**_−_ = (**j** − *z*_+_**N**_+_)/*z*_+_. This shows that the relevant diffusion coefficient for the transport of ions in the electrolyte is not the self-diffusion coefficient, which would be different from the diffusion coefficient of the anions, but the unique collective inter-diffusion coefficient. It cannot be determined directly by using, for example, nuclear magnetic resonance (NMR) measurements. However, there are simple approximations to determine the inter-diffusion coefficients from the measured self-diffusion coefficients [10,59].

For the experimental determination of the diffusion coefficient is is important to distinguish between the diffusion coefficient at vanishing electrical current and at vanishing gradient of the electrical potential. Only the coefficient at vanishing electrical current, i.e., the one obtained from Equation 44 is experimentally accessible. In DFT calculations the diffusion coefficient is written as a product of a thermodynamic factor *a_T_* and a kinetic coefficient [9–10]. The thermodynamic factor *a_T_* is the thermodynamic derivative of the chemical potential with respect to concentration *a_T_* = ∂μ/∂*c* and from the relation for the flux (Equation 44), we see that the kinetic coefficient is related to the determinant of the mobility matrix *B*.

By using the obtained expressions for the fluxes and the electric current we finally obtain the equation for the electric charge ρ_F_, the molar concentration of positive ions *c* := *c*_+_ and the entropy density:

[49]
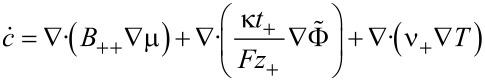


[50]
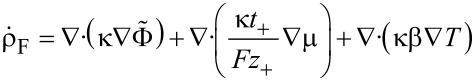


[51]
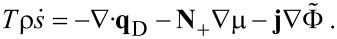


The electric charge density ρ_F_ is coupled to Coulomb’s law (Equation 10) and to the pressure equation (Equation 29). The entropy production is given by

[52]
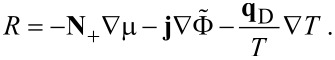


The different potentials Φ, 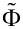 and φ have different physical meanings. Only the potential Φ is relevant for the calculation of electrical charges, e.g., in the double layer with the help of Coulomb's law (Equation 10). The chemical potentials μ, 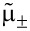 and μ_α_ are defined through Equation 26, once the materials law for the free energy density φ_H_ is defined.

#### Charge neutral systems

Most of the battery literature deals with locally charge-neutral systems, i.e., ρ_F_ = 0. This assumption is based on the observation that in a battery, under normal operating conditions, the potential differences required for charge separation exist only in a very thin layer around the active particles in the form of a double layer. To include the double layer, it is either necessary to solve the equations derived above without further assumptions on charge neutrality [27], or specific models for the double layer [60] or the form of the charge distributions around the active particles [61] are necessary. On a scale above a few nanometer one can safely assume, that the electrolyte is a charge neutral system, i.e., ρ_F_ = 0 and *c*_−_ = (*z*_+_/*z*_−_)*c*_+_. This also applies to the active particles. The shielding is even more effective due to the high mobility of the electrons in the active particles. If we only deal with a binary salt, we have *z*_+_ = *z*_−_ = 1, i.e., *c* = *c*_+_ = *c*_−_ at each point of the bulk of the electrolyte and the active particles. As a consequence it follows from Equation 26, that μ_+_ = μ_−_. The chemical potentials become a function of the temperature, the electric potential and the Li ion concentration, only. The pressure dependence can safely be neglected since it is only relevant in the double layer [27]. Also the possible dependence of the chemical potential on the electrical potential is usually not considered, since it is assumed that only short-ranged chemical forces are contributing to the chemical potential. No assumptions of this kind are necessary within the framework of rational thermodynamics. In the end, the form of the chosen materials law for the free energy φ_H_ (Equation 19) determines whether the chemical potential is dependent on the electrical potential or not. For example the chemical potential would depend on the electric field if the contribution of the polarizability of the electrolyte or the active particle to the free energy density depends not only on the electric field but also on ion concentrations. This would be the case in ion-conducting solid electrolytes. Neglecting these effects we may write

[53]
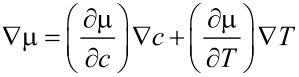


and introduce a new effective heat flux **Q**

[54]
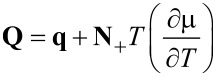


to obtain the constitutive relations Equation 44 in the form

[55]
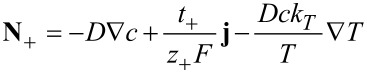


[56]
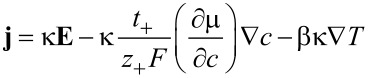


[57]
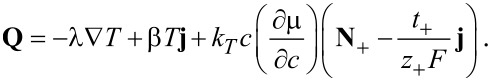


The diffusion coefficient *D*, the heat conductivity λ and the Soret coefficient *k_T_* are given by

[58]
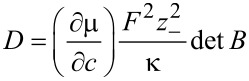


[59]
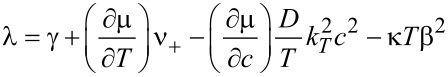


[60]
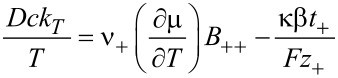


Using Equations 55–57, the expression for the entropy production (Equation 52) simplifies to

[61]
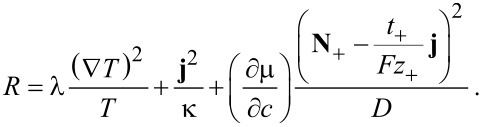


From the requirement of a strictly positive entropy production we can easily conclude that each term in Equation 61 has to be positive and obtain the well-known fact that the transport coefficients heat conductivity λ, electric conductivity κ and inter-diffusion coefficient *D* have to be positive. The equations of motion reduce to

[62]
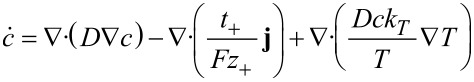


[63]
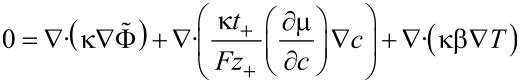


[64]
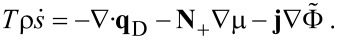


The equation for the temperature follows from the entropy equation (Equation 64) by using

[65]
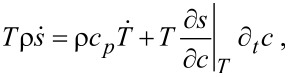


where *c_p_* is specific heat per unit mass. With the thermodynamic relation


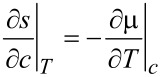


and the continuity equation (Equation 62) it was shown in [41] that the equation for the temperature is given by

[66]
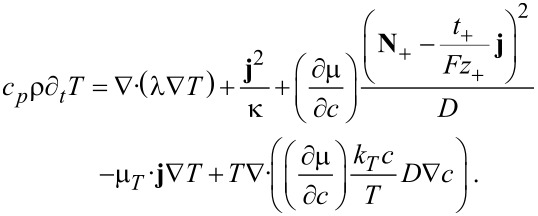


The Thomson coefficient μ*_T_* is defined by the so called Thomson relation 
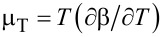
. Terms of order 
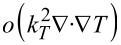
 are neglected. Temperature inhomogeneities are caused by four different types of heat sources and equilibrated by thermal conduction. The four heat sources are Joule’s heat, heat of mixing, Thomson effect and the Soret effect (in the order of their appearance in Equation 66). Since usually *k_T_* is very small we can safely neglect its contribution in most applications and obtain the approximated equation

[67]
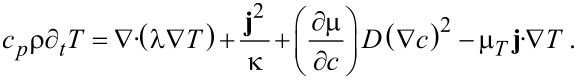


### Interface conditions

The equations derived above are valid in the electrolyte as well as in the active particles. The value and the physical mechanisms underlying the transport coefficients are different. Diffusion mechanisms in solids are different from those in electrolytes. Conduction in electrolytes is due to ion transport, but mostly of electronic nature in the active particles. These differences require different atomistic simulation techniques for the determination of the transport coefficients, but the form of the macroscopic equations is not affected by these differences. To couple the transport in the electrolyte and the active particles the formulations of interface conditions sometimes also called jump conditions are necessary. They are usually derived from the transport equations and models for processes on the interface, such as surface diffusion or electrochemical reactions on the interface, by using a pill box argument [39]. In this argument the transport equations are integrated over a small volume element, which includes the two sides of the interface. The thickness of the volume element is reduced to zero after the integration such that all volumetric contributions vanish compared to the surface contributions from the fluxes across the surfaces and the processes on the surface. To use the transport equations for the derivation of the interface conditions also guarantees that they are based on the same physical fields that are also used in the transport theory. In our case we need interface conditions that describe the intercalation and de-intercalation of ions, as well as the heat produced during this process.

#### Interface conditions for ionic flux and electric current

For cell-scale simulations, it is very difficult, if possible at all, to spatially resolve the processes involved in intercalation and de-intercalation numerically. Therefore it is common to use phenomenological global expressions for describing intercalation, although in reality this is a complex process that involves at least three steps: desolvation and adsorption on the external surface of the active particle, transfer of the Li ion from the external surface into the host material and shielding of the charges by lattice deformations and reorganization of the electronic charge density distribution. These elementary steps were identified experimentally [62–63] for the intercalation of Li in graphite. In this still very simple model no distinction is made between the various possible types of chemical bonds after intercalation. For instance, in [63] three different bonding states have been identified for the Li ions within various types of graphite. These different types of Li–graphite bonds are reflected by different chemical potentials and possibly different transition states for the respective intercalation step. For the actual intercalation step from the surface into the host material, it is possible to derive a Butler–Volmer-type expression from very general considerations by using only the mass action law and assuming the existence of a transition state [56,64–66]. A more realistic description of the intercalation will require the incorporation of at least the desolvation–adsorption step mentioned above. The extensions of the simple Butler–Volmer theory obtained through this can be easily incorporated in a generalized interface condition. In [64] the current density across the interface *i_se_* was derived from thermodynamic arguments, and a Butler–Volmer equation with modified expressions for the exchange current amplitude was obtained:

[68]
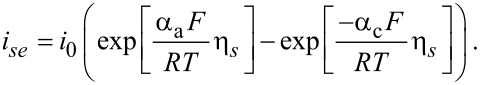


α_a_ and α_c_ with α_a_ + α_c_ = 1 are transfer coefficients, respectively. They quantify the fraction of the overpotential to the anodic and the cathodic charge transfer, respectively. It was shown in [64] that the sum of the transfer coefficients has to be 1 as a consequence of the law of mass action and requirement of positive entropy production during the transition from electrolyte to active particles. The overpotential is the difference between the electrochemical potentials of active particle and electrolyte, which vanishes in equilibrium:

[69]
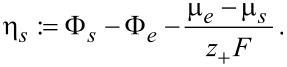


The same interpretation of the overpotential is also obtained in [56]. We can reformulate the overpotential (Equation 69) into the more conventional form

[70]
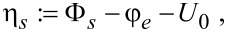


where *U*_0_ is the half-cell open circuit potential of the respective electrode relative to a Li metal electrode

[71]
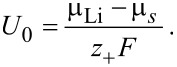


Also the electrochemical potential of the electrolyte φ*_e_* is defined relative to the chemical potential of Li metal:

[72]
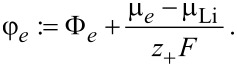


It is important to note, that as a consequence of introducing the open circuit voltage *U*_0_, there appears the difference of the electrical potential of the active particle and the electrochemical potential of the electrolyte in the definition of the overpotential. Both are measurable quantities, whereas the electrical potential of the electrolyte is not directly measurable. The amplitude *i*_0_ in Equation 68 deviates from the usual definition [67] due to thermodynamic reasons [64]

[73]
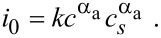


*k* is a reaction rate, which depends on the activation energy of the transition state for the ionic transfer from the electrolyte to the active particle. The full set of interface conditions for ionic flux and electric current follows from the assumption of continuity of ionic flux and electric current and the fact that only ions are transferred and, therefore, the whole electrical current across the interface is carried by the lithium ions. Side reactions leading to a degradation of the electrolyte [68] would lead to an additional electrical current due to electron transfer between active particles and electrolyte. With the normal **n** pointing from the solid into the electrolyte we obtain

[74]
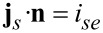


[75]
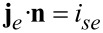


[76]
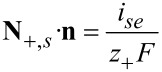


[77]
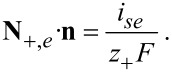


#### Thermal interface conditions

Under isothermal conditions, the values of the concentrations and potentials of the electrolyte and the solid particle at the interface can be determined from the interface conditions (Equations 74–76). If heat flux is considered the value of the temperature on the interface also has to be determined in order to be able to calculate the gradient of the temperature on the electrolyte side and solid side of the interface. The additional interface conditions can be derived by applying the pill box argument to the equations for the heat transport. We integrate the entropy balance equation (Equation 64) over an infinitesimal small volume element, which contains the whole thickness of double layer and use the jump discontinuity of the chemical potential and the electrical potential at the interface to obtain with Equation 74 and Equation 76

[78]
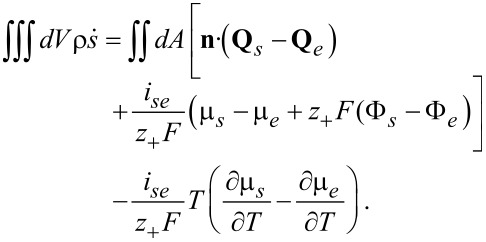


In the limit of vanishing thickness of the pill box, the left hand side of Equation 78 vanishes. If the weak temperature dependence of the chemical potential of Li metal is neglected, we finally obtain from Equation 57, Equation 71, and Equation 72 the expression in Equation 79.

[79]
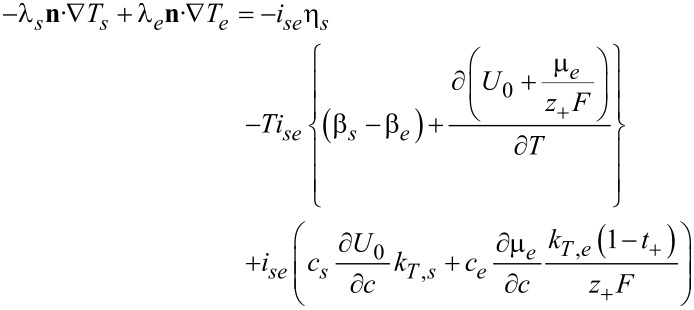


The transference number of Li ions in the active particle was set to zero, since the whole electrical current in the active particles is assumed to be given by the flux of electrons. Formulated differently, the mobility of electrons is much larger than the mobility of ions. With this condition for the jump in the gradients of the temperature, in addition to Equations 74–76, the values of all concentrations and potentials, and the temperature on the surface can be determined. On the right hand side of Equation 79 are the interfacial irreversible and reversible heat sources. The first is the irreversible interfacial Joule heating, followed by the reversible Peltier effect and the reversible Soret effect. The Peltier coefficient is defined by

[80]
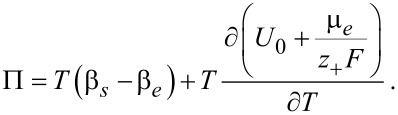


In addition to the partial derivative of the open-circuit potential [69] also the differences in the Seebeck coefficients of the two phases and the thermal derivative of the chemical potential of the electrolyte are contributing to the Peltier coefficient. It can either be measured directly [70] or may be deduced from measurements of the open-circuit potential, the Seebeck coefficients of the two phases [71] and the thermodynamic derivatives of the chemical potential of the electrolyte.

## Porous electrode theory: Volume averaging

The microscopic equations derived above allow one to study the spatial scale from about 10 nm up to a few hundred micrometer and are therefore suitable to analyze transport processes in the microstructure of a battery cell. The active particles and the electrolyte are treated as geometrically separate domains within this approach. Since the diameters of active particles range from a few micrometers down to tens of a nanometer, computationally resolving a cell of a few hundred micrometers thickness and lateral dimensions in the range of tens of a centimeter becomes very difficult, if possible at all. Therefore the preferred approach for simulating whole cells is the porous electrode theory, pioneered by Newman and coworkers [72–75]. A porous electrode theory for materials with phase transitions was formulated in [76]. To derive the porous electrode theory that corresponds to a given microscopic theory several methods of increasing complexity can be applied [77–79]. The most simple and straightforward approach is volume averaging. In this approach, the microscopic transport equations are integrated over a complex porous microstructure and the Gauss Theorem is used to derive the equations for the separate domains and the contribution of the interfaces to the transport [80]. This method does not constitute an analytical proof that the averaged solution of the microscopic equations does converge in a strict mathematical sense towards the solution of the averaged set of equations. Here, further analytical work or numerical convergence studies will be necessary.

If a quantity *A* is averaged over one phase, say the electrolyte phase *V_e_*, in a representative volume element (RVE) of volume *V* = *V_s_* + *V_e_*, we obtain

[81]
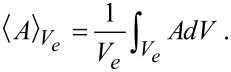


Central is the following theorem for the average of divergence terms, say 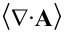:

[82]
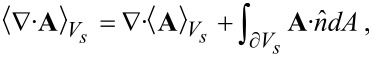


where *dA* is an infinitesimal area element and 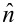 is the outward surface normal.

The volume-averaging method for isothermal systems is discussed in detail in [61,77–78]. For non-isothermal systems volume averaging was used to homogenize heuristic microscopic equations [45]. The terms proportional to 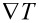 in the ionic flux and electric current will give rise to a contribution of the volume-averaged temperature gradient and of the jump of the temperature gradients at the interfaces between electrolyte and active particles. As will be shown below the contribution of these terms to the ionic fluxes and currents can be neglected compared to the volume-averaged gradients of the electrochemical potential and the jump of the electrochemical potentials at the interface. We therefore concentrate on the derivation of the volume-averaged temperature equation, consistent with the microscopic equation, derived above:

[83]
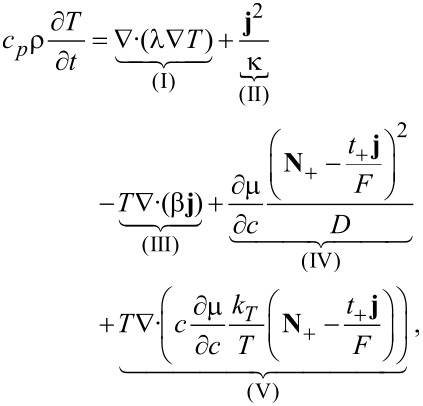


where the terms correspond to heat conduction (I), Joule heating (II), the Thomson effect (III), heat of mixing (IV) and Soret–Dufour effect (V). The technical details can be found in the Appendix. The final result for the volume averaged heat equation is

[84]
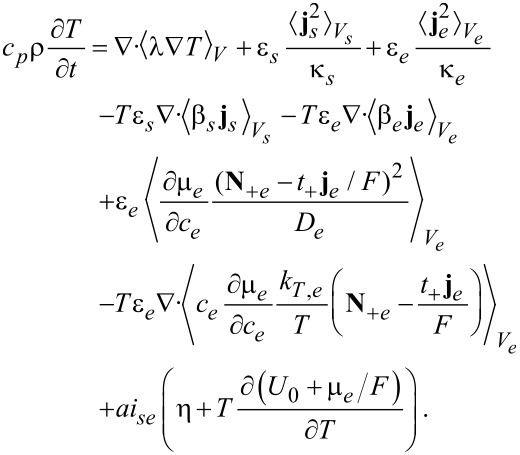


Interestingly, all surface terms due to the coupling of heat and ion transport (terms proportional to *k_T_*) from the Soret–Dufour effect and due to heat–current coupling from the Thomson effect cancel exactly with the corresponding terms from the contribution of the heat conduction. What remains is the surface Joule heating and the Peltier effect (last line of Equation 84).

## Comparison of microscopic and mesoscopic heat transport

There are many papers in literature, which present models and simulations of thermal effects on cell-level based on experimental investigations [44,49,81–84]. In this paper, we are not interested in fitting parameters to experimental results. Instead, we want to stress the intrinsic fundamental differences and similarities of models on two different spatial scales. Specifically, we will show that there are important phenomena on the microscopic scale which cannot be represented, in principle, through homogenized theories, but which may be very important for predictions of degradation phenomena.

In order to demonstrate these fundamental differences between microscopic and homogenized models, mostly based on porous electrode theory (we call it the mesoscopic approach), with respect to thermal aspects, we performed numerical computer simulations on a generic model system with two different micro structures and compared the results to meso-scale simulations of two corresponding setups.

### Simulation details

**Figure 1:**
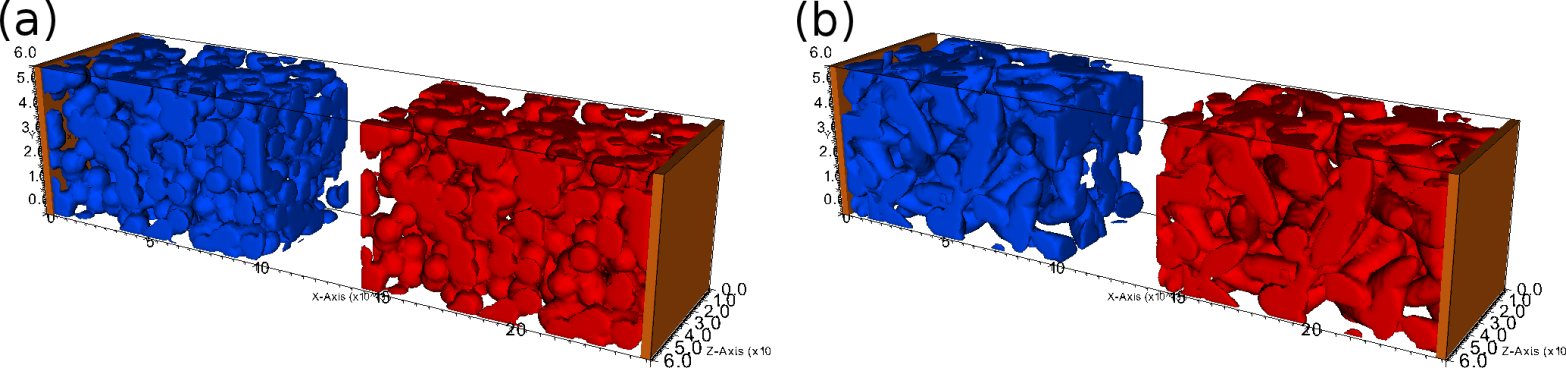
Geometry used for the microscopic simulation. It consists of an anode (blue), a cathode (red) and current collectors (brown). The space between particles and electrodes is filled with electrolyte. Geometry (a) consists of spherical particles of radius 5 μm, geometry (b) of a prolate spheroids of half-axes with 5 μm and 16.8 μm. The thickness of each electrode is 100 μm, the separator region 40 μm and the cross section area 60 × 60 μm^2^.

In order to demonstrate the qualitative features of the microscopic electrochemical model, no measured electrode structure was used but rather a computer-generated random geometry with typical properties. To simplify the geometry further both electrodes have an identical structure. Two cases with different base particles were considered: one with spherical active particles of radius 5 μm and one with prolate spheroids of random orientation with half-axes of 5 μm and 16.8 μm. In both cases the porosity ε was set to 0.5 such that the capacity of each electrode is equal. The geometries are shown in Figure 1. The left and the right electrode are the anode and the cathode, respectively. They are connected to current collectors through which electrons enter. Note that although electrodes are equal, their interface area with electrolyte differs slightly since they are attached to the collector plates on opposite sides. The simulation was set up such that the virtual cell is almost empty and a constant current was applied to charge the cell. Details on the parametrization are summarized in Table 1. Note that no temperature-dependence of the parameters was considered here. In particular, there is no contribution of ∂*U*_0_/∂*T* in the Peltier term (see Equation 80 and the last term of Equation 84), which might underestimate the contribution of this term.

**Table 1:** Summary of generic parameter set used for the microscopic simulations. Subscripts *s*, *e*, *cc*, *A*, and *C* denote solid, electrolyte, current collector, anode, and cathode, respectively. For the thermal equation this study chooses identical parameters for all materials species. Quantities marked with an asterisk differ in the meso-simulations. ^a,b^

quantity / unit	value	quantity / unit	value

*D_s,A_* / cm^2^s^−1^	10^−10^	*D_s,C_* / cm^2^s^−1^	10^−10^
σ*_s,A_* / S/cm	^*^10	σ*_s,C_* / S/cm	^*^0.38
σ*_cc,A_* / S/cm	^*^10	σ*_cc,C_* / S/cm	^*^0.38
*k* / A cm^2.5^ mol^−1.5^	0.002	*k* / A cm^2.5^ mol^−1.5^	0.2
*c*_max,_*_A_* / mol/cm^3^	24681 · 10^−6^	*c*_max,_*_C_* / mol/cm^3^	23671 · 10^−6^
*c_0,A_* / mol/cm^3^	2639 · 10^−6^	*c_0,C_* / mol/cm^3^	20574 · 10^−6^
*c_0,e_* / mol/cm^3^	1200 · 10^−6^	*t*_+_ / 1	0.39989
*D_e_* / cm^2^s^−1^	^*^1.622 · 10^−6^	κ*_e_* / S/cm	^*^0.02
λ / W/(cm K)	0.006	*c_p_* / J/(kg K)	4180
ρ / kg/cm^3^	0.001	β / V/K	0.0002
*k_T_* / 1	1	*T*_0_ / K	298
*i*_appl_ / A/cm^2^	0.00318		

^a^*U_0,A_*(*soc*)/V = −0.132 + 1.41 × exp(−3.52 × *soc*) ^b^*U_0,C_*(*soc*)/V=4.06279 + 0.0677504 × tanh(−21.8502 × soc + 12.8268) − 0.105734 × ((1.00167 − *soc*)^−0.379571^ − 1.576) − 0.045 × exp(−71.69 × *soc*^8^) + 0.01 × exp(−200 × (*soc* − 0.19))

To solve the PDE system of the thermal micro-model Equation 62, Equation 63 and Equation 66 for the unknown quantities concentration *c*, potential Φ and temperature *T* the finite-volume method [85] was employed. To this end the simulation domain of Figure 1 is discretized into a regular grid of cubic control volumes (CVs). In this discretization the unknown quantities are only defined in the center points of the CVs. Separate integration of the transport equations over the volume of each CV *i* and application of the Gauss theorem turn the PDE system into a large algebraic system that can be solved numerically by a computer. Time evolution is discretized by using the simple backward Euler scheme with a time step of 20 s. One time step consists of the following three steps:

1. Due to the strong coupling between concentration and potential the system Equation 62 and Equation 63 is solved monolithically.
2. Solution of the temperature system Equation 66.
3. In order to improve accuracy and maintain conservation properties step 1 is repeated with the new temperature.

Since the equations describe a nonlinear PDE-system a nonlinear solver must be used for each of the above steps. We employ a simple Newton algorithm in combination with the algebraic multigrid solver SAMG [86] to deal with the nonlinearities. This approach is implemented in the software BEST [87] (based on the CoRheoS framework [88]), which was applied to perform the simulations.

**Figure 2:**
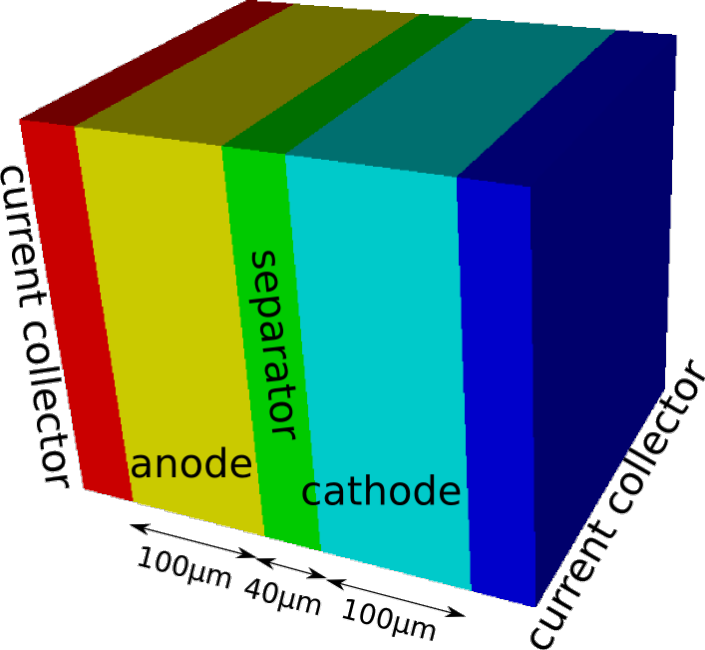
Geometry for mesoscopic simulations with the porous-electrode model.

The geometry used for the mesoscopic simulations is shown in Figure 2. Except for the current collectors that have now a thickness of 40 μm the thicknesses of electrodes and separator are the same as in the case of the microscopic scale. The lateral dimensions were increased to 260 μm but the applied current was scaled proportionally. The parametrization was chosen such that meso- and micro-simulations can be compared (Table 2). To this end the effective transport properties required in the meso-case instead of bulk values are computed from the micro-structure by using the software GeoDict [89].

**Table 2:** Parameters used for the mesoscopic simulations that differ from the case of microscopic simulations (cf. Table 1). Due to the different structures of spherical and ellipsoidal micro-geometries the effective transport parameters are different. Subscripts *e*, *s*, *AC* and *Sep* denote electrolyte, solid, anode/cathode and separator, respectively.

quantity / unit	value (sphere)	value (ellipsoid)

*D_e,AC_* / cm^2^s^−1^	0.474 · 10^−6^	0.438 · 10^−6^
*D_e,Sep_* / cm^2^s^−1^	1.622 · 10^−6^	1.622 · 10^−6^
σ*_s,A_* / S/cm	1.246	1.82
σ*_s,C_* / S/cm	0.047	0.069
κ*_e,AC_* / S/cm	0.00584	0.0054
κ*_e,Sep_* / S/cm	0.02	0.02
*r_s_* / μm	4.89	5.93

As described in the previous section, the meso-model used here is a 3D + 1D model, i.e., three spatial dimensions for the unknown quantities electrolyte concentration, electrolyte potential, solid potential and temperature and, in each electrode CV, another virtual dimension for the concentration within a representative spherical particle (solid concentration) to mimic the diffusion into the active material. As in the micro-model a finite volume discretization is used for both the 3D cell geometry and the 1D domain representing a microscopic particle. Here, each 3D electrode voxel contains its extra dimension for the representative particle which is spatially discretized into 10 control volumes. The solution process of each time step with fixed step size of 15 s is as follows:

1. Solve the coupled system for the unknown quantities electrolyte concentration, electrolyte potential, solid potential in the 3D domain.
2. Solve the solid particle diffusion problems for the unknown solid concentration in the 1D domain for each electrode voxel individually.
3. Solve the temperature equation for *T* (in the 3D domain).
4. Repeat step 1 to improve accuracy and maintain conservation properties.

Steps 1 and 3 are solved implicitly with a simple Newton method and an algebraic multigrid solver [86] whereas step 2 uses an explicit forward Euler discretization based on the same time step and is thus fast to solve. However, due to the time-step limitations for explicit schemes the step size here can automatically decrease based on the given parameters to ensure stable convergence.

Despite the fact that on the microscale the basic particles are spherical and ellipsoidal, the porous electrode theory mimics the solid diffusion process always by a sphere of a certain radius. This radius *r* is related to the specific interface area *a* and the electrode porosity ε by *a* = 3ε/*r*. Here we chose the approach to determine *a* from the micro-geometry and compute the corresponding radius *r* but we note that there are also other reasonable approaches to fix *r*.

## Results and Discussion

**Figure 3:**
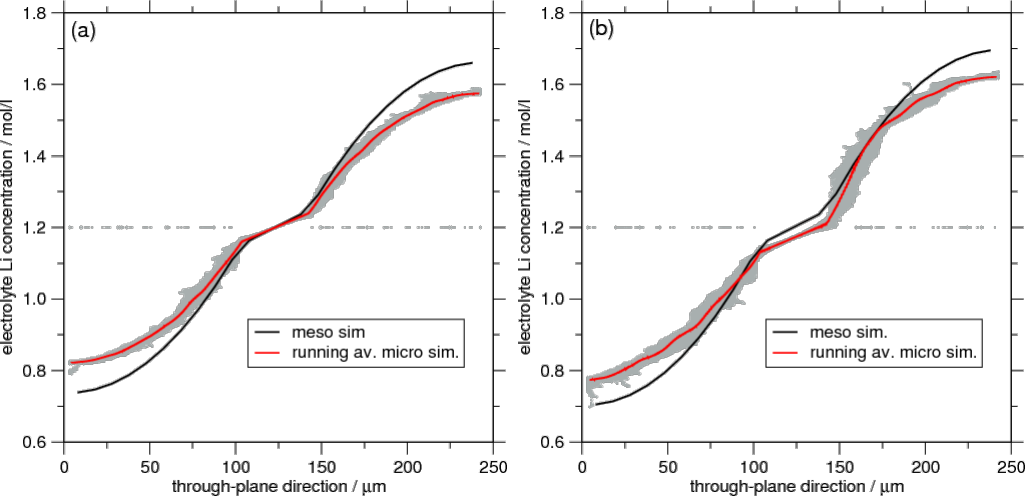
Distribution of electrolyte concentration in dependence of position in through-plane direction at a capacity ratio of 0.42 for (a) spherical particles and (b) prolate spheroids. Each panel compares results of the micro-simulation with a corresponding meso-scale simulation.

The simulation yields a three-dimensional field of lithium ion concentrations. Of interest is, for instance, the ion concentration in through-plane direction. A projection of the Li concentration of the electrolyte phase onto this axis is shown in Figure 3. The data from the microscopic simulations shows considerable scatter reflecting the inhomogeneous, random structure. There are even CVs that remain at their initial concentration of 1.2 mol/L since they are surrounded completely by active particles. Due to the electroneutrality condition they have to stay at their initial concentration. The porous electrode approach treats the complete electrode region as homogeneous effective medium. For this reason (and of course due to the application of symmetric boundary conditions) the concentration profile does not show any scatter and agrees reasonably well with the microscopic average. However, a quantitative agreement is only obtained in the separator region for the sphere-based micro-geometry. Especially in the electrodes far away from the separator there is a deviation of about 5%. Since the porous electrode model is in some sense a simplification of the microscopic approach its results must be scrutinized more carefully. The concentration within the electrolyte depends on the effective parameters for diffusion and ionic conductivity. In this study they were computed by performing a simplified transport simulation in the same microstructure that was used for the microscopic battery simulations. Due to the limited geometry size that was used it is likely that the effective parameters are not very exact. Furthermore, it is questionable whether the meso-approach is justified at all here since we use it on a similar scale. As pointed out in [79] the necessary condition of scale separation for using homogenization is not fulfilled in real lithium ion batteries. Very often electrodes have a thickness of the order of only ten particle diameters, which is not sufficient to justify the assumption of scale separation. Therefore it seems reasonable that for the ellipsoidal base particles the concentration in the separator region does not match well because in this case also the representative sphere of the meso-model is different from the actual micro-particle (ellipsoid). Thus, it is important to determine the effective properties as well as the representative particle size with great care. This is very relevant, for instance, for a prediction of the limiting current, at which the electrolyte is locally depleted of ions.

**Figure 4:**
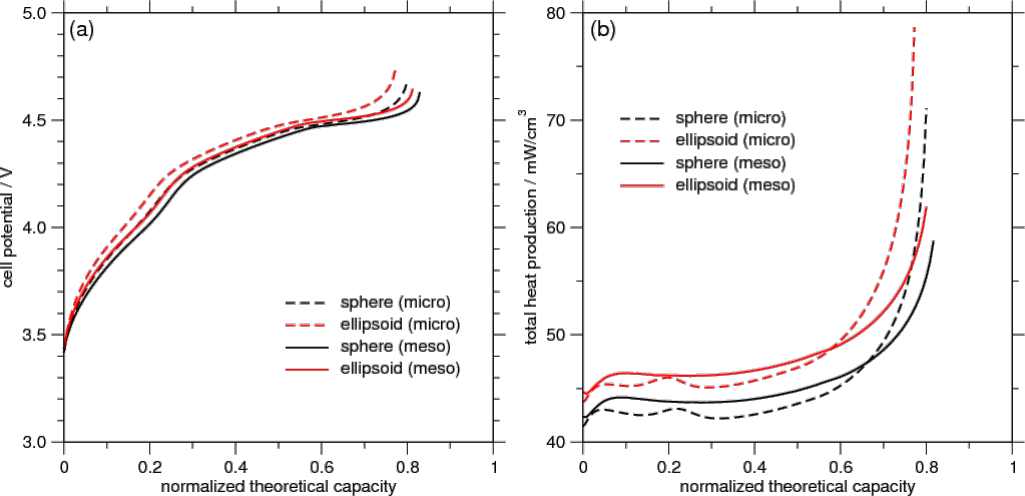
(a) Comparison of cell potential during charging simulations on micro- and meso-scale for the two base particles considered here, sphere and ellipsoid. (b) Comparison of heat production shown in a similar way as in (a).

The concentration distribution within the active material is indirectly expressed through the cell potential (differences between current collector potential at cathode and anode). For the four simulation cases the cell-potential is shown in Figure 4a. Comparing the microscopic cell potentials it is interesting to note the higher voltage for the ellipsoidal geometry compared to the spherical geometry. This is explained by the different interface areas: While the ellipsoidal micro-geometry has an interface area of 0.18 mm^2^, it is 0.22 mm^2^ for the one based on spheres. Since in both cases the same current is applied a higher overpotential (and therefore cell potential) is required for smaller interface areas. Although the meso-simulation shows the same behavior the corresponding micro and meso simulations differ slightly. Since the lithium diffusion within active particles is modeled in the meso-case only by single, representative particles, the exact influence of the actual interface shape and connectivity between particles is neglected. Thus, it cannot be expected that both methods show a better agreement without more careful adjustment of parameters, in particular of the radius *r* in the meso-model. However, even then it is not clear that this will lead to the same results also in other application scenarios, e.g., with a different applied current.

Thermal effects are of particular interest in this model comparison. Because the meso-model neglects structural details the current and the ion flow are very different compared to the microscopic model. In the latter, the current can be strongly localized due to the connective properties of the electrode structure. Thus, it is very surprising that a comparison of the total heat power per volume is very similar for both simulation approaches (see Figure 4b). Additionally, as before, we recognize a difference of heat production between spherical and ellipsoidal base particles. To understand this we need to analyze the different sources of heat.

**Figure 5:**
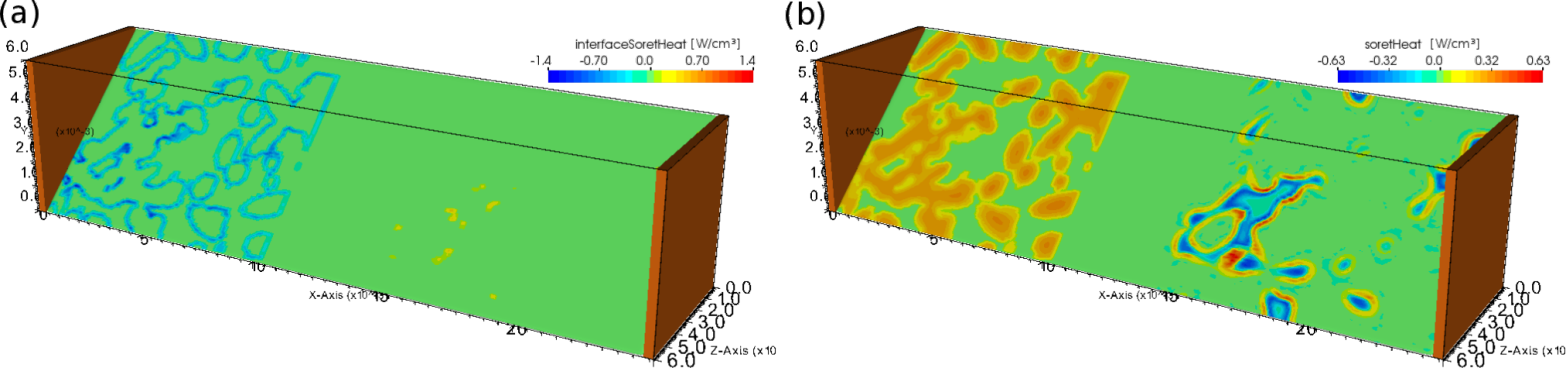
Cut through the ellipsoid-based micro-geometry showing heat production at a normalized capacity of 0.5 by (a) the interface Soret effect and (b) the bulk Soret effect.

**Figure 6:**
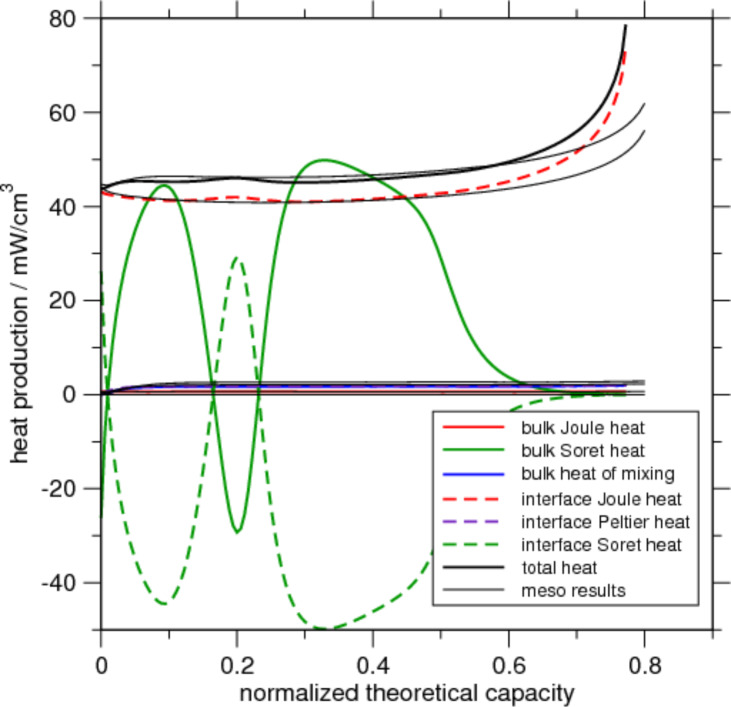
Heat production for ellipsoid-based micro-structure due to different heat sources. Thick lines show the results from the micro-simulation while the thin black lines are the results of the corresponding meso-simulation.

The microscopic model allows one to study the heat sources with spatial resolution. As an example we show in Figure 5 the heat production caused by the Soret effect in bulk and at the interfaces. Since it is a reversible heat source, positive and negative contributions dependent on the local current directions are possible. It is clearly visible that heat sources can be strongly localized depending on the micro-structure. This is true also for other sources of heat. By computing the total power per volume for the different heat sources their relative magnitudes can be compared as function of state-of-charge during charging. Figure 6 shows the result for the microscopic simulation with ellipsoidal base particles. A very interesting feature is the time-dependence of the Soret heat: Although it oscillates strongly with a large magnitude and is very inhomogeneously distributed in space (cf. Figure 5) the total interface and bulk contributions cancel each other exactly such that the Soret effect is of no importance for the total heat production in this case. Responsible for the shape of the Soret curves is the derivative of the open-circuit potential (OCV) (cf. last terms in Equation 57 and Equation 79). After averaging those terms vanish for the mesoscopic model (cf. second last term of Equation 84). What remains is the divergence of the purely diffusive ion flux which is very small in the separator and has opposite signs in the electrodes. In a symmetric setup such as the one studied here with sufficiently small temperature gradients these (the last terms in Equation 57 and Equation 79) basically cancel. That is why the Soret contribution in the meso-simulations is of the order of 10^−5^ mW/cm^3^.

We realize from Figure 6 that the only significant contribution to the total heat production comes from the irreversible Joule heat created at the interface between active particle and electrolyte, i.e., basically the product of overpotential and Butler–Volmer current η*i_se_* (cf. last line of Equation 84). We further note the good agreement between micro- and meso-scale simulations. In particular the interface Joule heat as most relevant heat source can be captured in the meso-model quite well, although there is a deviation at the end of the charging process. Similar to the cell potential (cf Figure 4) also the interface Joule heat crucially depends on the overpotential. Therefore, the same reason as discussed before explains the difference.

**Figure 7:**
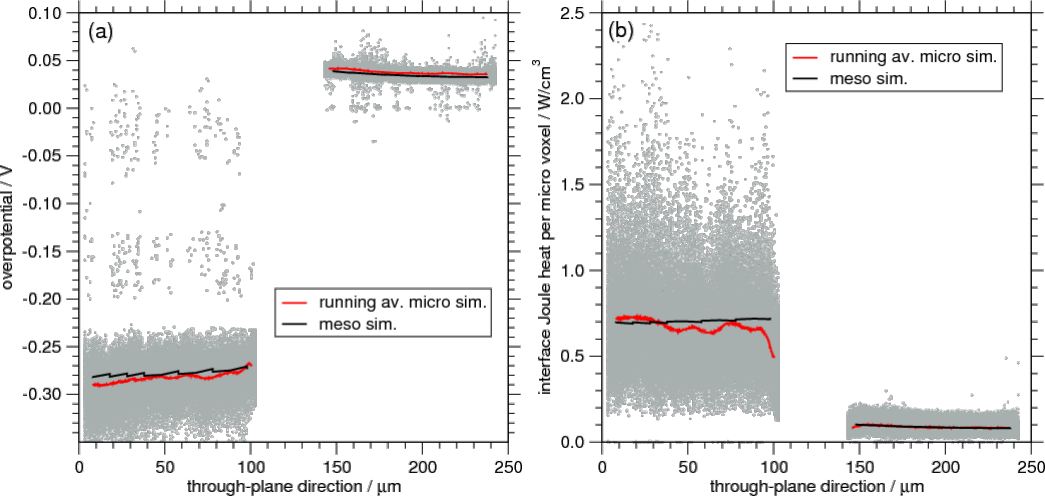
(a) Spatial distribution of the overpotential in through-plane direction for the ellipsoid-based microstructure at a capacity ratio of 0.42. Due to the strong variation of the data in the microscopic simulations (grey), a running average (red) is compared to the overpotentials of the mesoscopic simulations (black). (b) Similar as in (a) we compare the Joule heat at the interface between active particles and electrolyte.

From the above discussion one would expect that the overpotentials of micro- and meso-model are the same. In Figure 7a we study the overpotential at a fixed time as a function of the position in the through-plane direction. Comparing the overpotentials for each CV in the microscopic case (grey dots) a very large scatter is observed that is larger for the anode (left). The anode has a lower rate constant than the cathode and hence a larger overpotential. The large scatter is of course an expression of the complex microstructure and for better comparability we compute a running average of the data (red curve). In the homogeneous mesoscopic simulation the overpotential curve is, by construction, much smoother and it agrees well with the microscopic data. Due to the strong relationship between the interface Joule heating with the overpotential the same holds for this heat source shown in Figure 7b. From both figures it can thus be concluded that the meso-approach is capable of reproducing the average or global heat power. However, one should note that this is because it is the interface Joule heat that plays the dominant role. So as long as the geometry is such that overpotential and Butler–Volmer current density can be described well on the mesoscopic scale the respective simulation can compute the correct amount of heat. If other sources of heat that rely on the actual distribution of current or ion flux become more important, either due to different material parameter combinations or geometric properties, it is unlikely that a meso-simulation can reproduce the results of the full microscopic approach.

**Figure 8:**
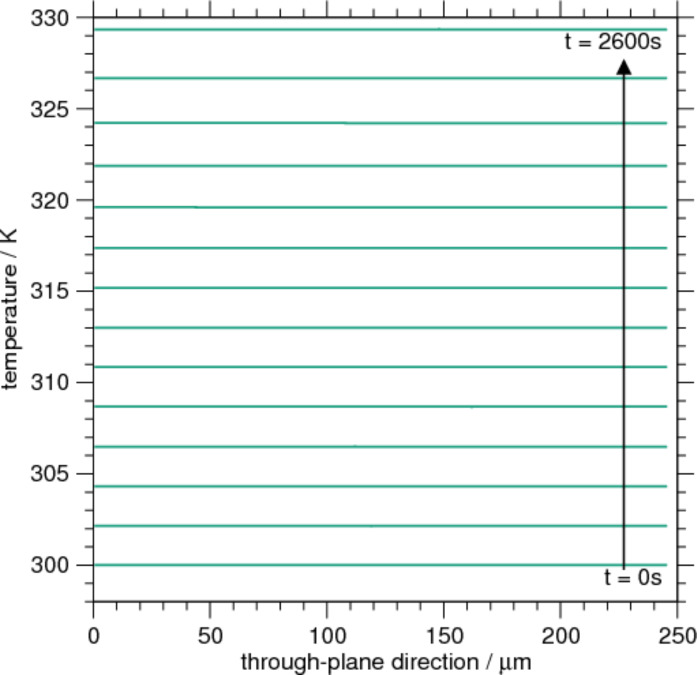
Spatial variation of temperature in through-plane direction for the microscopic ellipsoid case.

Many processes, e.g., the intercalation rate or degradation effects, depend on the local temperature. Having realized from Figure 5 and Figure 7 that heat sources are inhomogeneously distributed in the realistic microscopic geometry it is of interest whether the resulting temperature exhibits a similar behavior. Therefore Figure 8 shows the cell temperature as a function of the through-plane position for different times during the charging. There is no spatial temperature variation visible (even on changing the scale), only a global increase of *T*. This is not surprising since the thermal diffusivity λ/*c_p_*ρ = 0.0014 cm^2^/s and the cell thickness of 0.00246 cm lead to thermal diffusion time scale of less than 0.5 s. The energy produced in any point inside the cell is thus spread out very quickly such that no appreciable gradient can develop. This is particularly true for the strongly localized interface Joule heat sources.

One finding of the presented numerical experiments is that it is mainly the Joule heating at the interface between active material and electrolyte that determines the amount of heat production. However, we have to repeat in this context that a temperature dependence of the parameters was not considered here, which in particular affects the reversible Peltier term (Equation 80). Some studies claim this term to be also of relevant magnitude at least for low currents [50]. A reliable experimental determination of ∂*U*_0_/∂*T* is, therefore, an important prerequisite for further computations including this effect, but they are not trivial since *U*_0_ only very weakly depends on *T*.

In summary, we found that the mesoscopic simulation approach was able to quantitatively reproduce the amount of energy produced during charging with a constant current from the microscopic approach. Although this was at first surprising since the scatter of concentrations, potentials or currents in the meso-model is much weaker, this result could be explained by the fact that there is only one dominant source of heat, namely Joule heating at particle–electrolyte interface. This heat term does only depend on overpotentials and intercalation currents that are reproduced in this approach reasonably well. As long as the solid-diffusion behavior, which influences the overpotential, is captured correctly and as the interface Joule heating is the dominant effect one can expect that the thermal results of the meso-model are as trustworthy as a full microscopic simulation. While the former was easily obtained in this study because the basic particles were of very simple spherical or spheroidal shape of equal size the latter might be different for other cases (e.g., other materials with different parameters or geometries). When the active particles exhibit a broad size and shape distribution, as in reality, more care is necessary to adjust their size and the solid diffusion constant in the meso-model. That such an adjustment would be sufficiently universal to allow for predictive simulations for a wide range of application scenarios (e.g., different load cases) can be doubted. Other studies state at least that the mesoscopic simulation approach underestimates the total amount of generated heat [50] when compared to the micro-approach. The latter takes structural influences into account and is thus less dependent on parameter adjustments or data fitting. Therefore more realistic results can be expected from the micro-approach. On the other hand, micro-scale simulations are computationally much more expensive if a reasonable realistic and representative micro-structure (e.g., from measurements) is used.

## Conclusion

Fully seamless multiscale simulations from atomistic to cell scale are not yet possible nowadays. There are unresolved issues on each scale, although tremendous progress for electrochemical storage applications have been achieved. In the main part of our paper, we addressed the problem on how the continuum scale, which stretches for battery cells from the nanometer to the centimeter scale can be systematically be brought in contact with the atomistic scale. We believe that systematic methods from non-equilibrium or rational thermodynamics may well be the tool to establish the connection with MD and DFT simulations, if all these methods are combined with methods from statistical mechanics to ensure the proper averaging strategies for obtaining the transport coefficients and thermodynamic derivatives [90]. In addition to giving a short overview over the available atomistic scale simulation tools, we concentrated on the problem of systematically deriving the coupled equations for ion, charge and heat transport in insertion batteries on the particle and pore resolved nano- and microscales of electrodes and upscaling these equations to the cell scale. In the derivation we presented a careful discussion on how the resulting form of the transport equations is influenced by the choice of the macroscopic fields and how the seemingly different models in the literature can be transformed into each other. The accuracy of the upscaling procedure was investigated numerically by comparing a fully 3D microstructure resolved model of a battery cell with the homogenized or upscaled representation of this cell. For the chosen microstructure the numerically thermal and voltage behavior of the cell under load was found to be astonishingly similar to the directly simulated results of the analytically averaged equations. This result cannot be considered to be true in general. We except that the differences can be much bigger for less homogeneously chosen microstructures. But even for the investigated homogenous microstructures huge fluctuations (larger then 100%) around the average for, e.g., overpotentials and Joule heating terms are found. As a consequence we may conclude that the probability for the occurence of degradation phenomena may be hugely underestimated. For example the occurence of plating depends on the local potential at the interface of the electrolyte and active particles. Since overpotentials are strongly underestimated, a porous electrode model cannot accurately detect the sites in the battery cell where, e.g., plating is likely to occur, that is where the total potential drops below the redox potential of Li/Li^+^. The same is to be expected for the initiation for thermally induced degradation of the electrolyte due to the underestimation of the local heat production. Although the temperature is basically constant on the microstructural scale, it is, in addition to the temperature, the locally available energy on very short time scales, which is relevant for overcoming reaction barriers of degradation mechanisms. Therefore, the local fluctuations in the heat sources may be relevant for the identification of critical structural properties of electrodes. For the design of optimal cells a combination of cell-scale simulations with pore-resolved simulations will be necessary to identify the best global behavior as well as the optimal structure on the nano-and micrometerscale, once the optimal material parameters have been identified with DFT- and MD- simulations.

## Appendix

To obtain the volume-averaged transport equation for heat (Equation 84), the volume averaging technique is applied to each term of Equation 83, where the convention is adopted that the normals always point from solid to electrolyte phase.

### Heat conduction

[85]
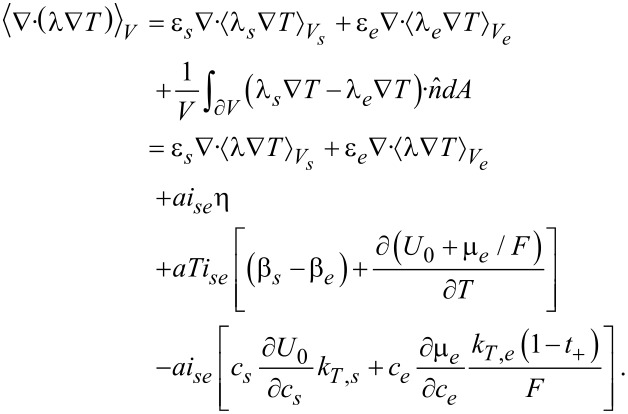


In the last step the microscopic expression for the thermal interface conditions was applied. Here and in the following *a* = *A*/*V* denotes the specific surface area.

### Joule heating

[86]
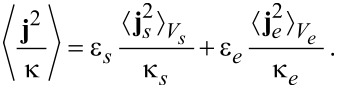


### Thomson effect

[87]
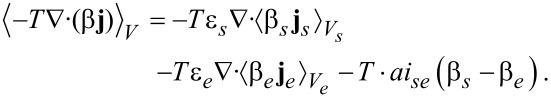


### Heat of mixing

[88]
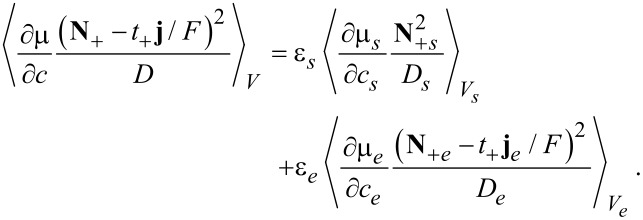


In the first term on the right hand side the transference number in the solid is zero.

### Soret–Dufour effect

The mathemiatical expression for the Soret–Dufour effect is given in Equation 89. Adding up all the contribution and assuming that there is no direct inter-particle transport of ions, i.e., 
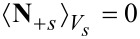
, we obtain the volume-averaged heat equation (Equation 84).

[89]
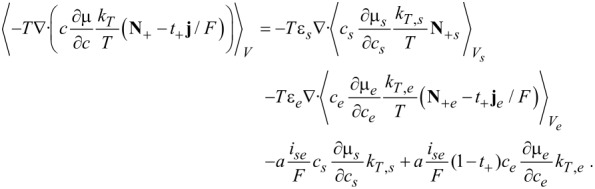


## Acknowledgements

The thermal extension of BEST was financially supported by the German Federal Ministry of Education and Research (BMBF) through the project ”TopBat”, grant 16N12529.
